# The relation of poor mastication with cognition and dementia risk: a population-based longitudinal study

**DOI:** 10.18632/aging.103156

**Published:** 2020-04-30

**Authors:** Christina S. Dintica, Anna Marseglia, Inger Wårdh, Per Stjernfeldt Elgestad, Debora Rizzuto, Ying Shang, Weili Xu, Nancy L. Pedersen

**Affiliations:** 1 Aging Research Center, Department of Neurobiology, Care Sciences and Society, Karolinska Institute and Stockholm University, Stockholm, Sweden; 2 Department of Dental Medicine, Karolinska Institute, Stockholm, Sweden; 3 Academic Centre of Geriatric Dentistry, Karolinska Institute, Stockholm, Sweden; 4 The Stockholm Gerontology Research Center- Äldrecentrum, Stockholm, Sweden; 5 Department of Epidemiology and Biostatistics, School of Public Health, Tianjin Medical University, Tianjin, China; 6 Department of Medical Epidemiology and Biostatistics, Karolinska Institute, Stockholm, Sweden; 7 Department of Psychology, University of Southern California, Los Angeles, CA 90089, USA

**Keywords:** mastication, cognitive decline, dementia, cohort study, longitudinal

## Abstract

We investigated the effect of poor masticatory ability on cognitive trajectories and dementia risk in older adults. 544 cognitively intact adults aged ≥50 were followed for up to 22 years. Cognitive domains (verbal, spatial/fluid, memory, and perceptual speed) were assessed at baseline and follow-ups. Dementia was ascertained according to standard criteria. Masticatory ability was assessed using the Eichner Index and categorized according to the number of posterior occlusal zones: A (all four), B (3-1), and C (none).

At baseline, 147 (27.0%) participants were in Eichner category A, 169 (31.1%) in B and 228 (41.9%) in C. After the age of 65, participants in Eichner category B and C showed an accelerated decline in spatial/fluid abilities (β: -0.16, 95% CI: -0.30 to -0.03) and (β: -0.15, 95% CI: -0.28 to -0.02), respectively. Over the follow-up, 52 incident dementia cases were identified. Eichner categories B or C were not associated with an increased risk of dementia, compared to category A (Hazard Ratio [HR]: 0.83, 95% CI: 0.39 to 1.76 and HR: 0.63, 95% CI: 0.30 to 1.29, respectively).

Poor masticatory ability is associated with an accelerated cognitive decline in fluid/spatial abilities, however it was not related to a higher risk of dementia.

## INTRODUCTION

The global population is growing older and one consequence of this is an increase in the prevalence of dementia, which is now the greatest global challenge for health and social care [1]. Meanwhile, older people are at a high risk of oral problems (such as periodontal disease, orofacial pain and the loss of teeth) [2]. The prevalence of edentulism (complete tooth loss) is 4.1% and peaks at around 25% in adults 75 to 79 years old [3].

Several studies have shown that tooth loss is associated with steeper global cognitive decline [4, 5], an increased risk for cognitive impairment [6–8] and dementia [9], as well as brain atrophy in cognitively normal older individuals [10]. One cross-sectional study found that jaw mobility, bite strength and complaints about masticatory function were associated with variation in episodic memory and executive function [11]. Moreover, emerging evidence suggests that the mechanism behind the association between tooth loss and cognitive decline is linked to reduced mechanical sensory input from poor mastication resulting from the loss of teeth [4, 10].

Mastication can be assessed objectively in terms of bite force, jaw mobility, and number and pattern of occlusal contacts (contact of opposite teeth in upper and lower jaw), and may be closely related to the number and distribution of remaining teeth [15]. Posterior occlusal contact of the remaining dentition has been reported as a marker of poorer masticatory ability [16–18]. The number of posterior occlusal contacts has been associated with cognitive function and a lack of posterior occlusal support influences cognitive decline to a greater extent than the number of teeth alone [19]. So far, only one longitudinal study investigated the relationship between posterior occlusal support and cognitive decline, showing that a lack of posterior occlusal support predicted global cognitive decline [20]. However, no studies have yet investigated the relation of mastication to trajectories in different cognitive domains and dementia risk.

In this study, we aimed to 1) examine the association between poor masticatory ability (reduced posterior occlusal support) and cognitive trajectories in different domains; and 2) investigate whether poor masticatory ability may increase the risk of dementia, using longitudinal data from a population–based study with up to 22 years of follow-up.

## RESULTS

### Characteristics of the study population

Over the follow-up period, 99 (18.2%) participated in all the waves, 44 (8.1%) participated in at least two follow-ups, 1 (0.2%) participated only at study entry, and 400 participants (73.5%) died. The median follow-up time was 10 years, IQR= 16-3. The number of participants in Eichner category A was 147 (27.0%), in category B 169 (31.1%), and in category C 228 (41.9%). Compared to those in Eichner category A, those in B and C were older and had lower education, while participants in Eichner category C consumed less alcohol, had lower education and childhood SES, and had higher proportions of belonging to the early birth cohort, having heart disease, hypertension, wearing prosthetics, and periodontal disease. Performance in all cognitive domains were lower for those with Eichner category B and C compared to A, except for verbal ability which was only significantly different between category A and C (Table 1). During the study period, 78 (53.1%) died in Eichner category A, 116 (68.6%) in category B, and 206 (90.4%) in category C.

**Table 1:** Characteristics of the study population at baseline by Eichner Index categories (*n*= 544).

**Characteristics**	**Category A, n=147 (27.0%)**	**Category B, n= 169 (31.1%)**	**Category C, n= 228 (41.9%)**	***P value***
Age /years	60.1 (±7.9)	64.6 (±8.3)^a^	69.9 (±7.6)^a,b^	<0.001
Female sex	80 (54.4)	98 (58.0)	136 (59.7)	0.604
Education				
Low	59 (40.7)	98 (60.1)^a^	172 (77.5)^a,b^	<0.001
High	86 (59.3)	65 (39.9)^a^	50 (22.5)^a,b^	
Hypertension	54 (36.7)	80 (47.3)	121 (53.1)^a^	0.008
Heart disease	10 (6.8)	21 (12.6)	43 (18.9)^a^	0.004
Diabetes	7 (4.8)	13 (7.7)	20 (8.8)	0.341
Cerebrovascular disease	0 (0.0)	3 (1.9)	4 (2.0=	0.254
Any *APOE* ε4	43 (31.6)	44 (28.6)	63 (31.3)	0.812
BMI (kg/m^2^)	25.0 (±3.6)	25.8 (±4.5)	25.9 (±3.8)	0.105
Smokers	71 (48.0)	73 (42.0)	109 (43.8)	0.631
Current	37 (25.9)	33 (20.0)	56 (25.3)	
Past	34 (23.8)	37 (22.4)	46 (20.8)	
Never	72 (50.4)	95 (57.6)	119 (53.9)	
Alcohol drinkers	135 (91.8)	142 (84.0)	177 (77.6)^a^	0.001
Childhood SES	0.6 (±2.6)	-0.1 (±2.3)	-0.7 (±2.2)^a^	<0.001
Birth cohort				
Early born 1886-1925	46 (31.3)	91 (53.9)^a^	180 (79.0)^a,b^	<0.001
Late born 1926-1958	101 (68.7)	78 (46.2)^a^	48 (21.1)^a*^	
Gingivitis				
No	113 (76.9)	132 (78.1)	192 (85.0)	0.094
Sometimes/Yes	34 (23.1)	37 (21.9)	34 (15.0)	
Periodontitis	14 (9.7)	26 (15.5)	68 (31.9)^a,b^	<0.001
Prosthesis				
None	96 (65.3)	83 (49.1)^a^	21 (9.2)^a,b^	<0.001
Half	0 (0.0)	0 (0.0)	185 (81.1)^a,b^	
Whole	51 (36.7)	86 (50.9)^a^	22 (9.7)^a,b^	
Cognitive performance				
Verbal ability	55.0 (±8.7)	52.5 (±8.3)	49.2 (±8.7)^a,b^	<0.001
Memory	56.03 (±9.4)	51.5 (±9.4)^a^	48.8 (±9.9)^a^	<0.001
Spatial/fluid abilities	56.4 (±9.4)	51.8 (±8.0)^a^	47.9 (±9.2)^a,b^	<0.001
Perceptual speed	57.2 (±9.0)	52.3 (±8.6)^a^	46.5 (±9.2)^a,b^	<0.001
General cognitive score^c^	57.5 (±9.1)	52.6 (±7.9)^a^	47.2 (±9.0)^a,b^	<0.001

Abbreviations: BMI= Body Mass Index; *APOE*= apolipoprotein; SES= Socioeconomic status.

Data are n (%) or mean (±SD).

^a^Bonferroni pairwise comparison (reference= Eichner Index A).

^b^Significant difference Eichner Index B vs C.

^c^Cognitive component score (T-score, mean= 50, standard deviation= 10) from cognitive tests of verbal ability, memory, spatial/fluid abilities, and perceptual speed.

### Eichner categories in relation to cognitive decline

**Figure 1:**
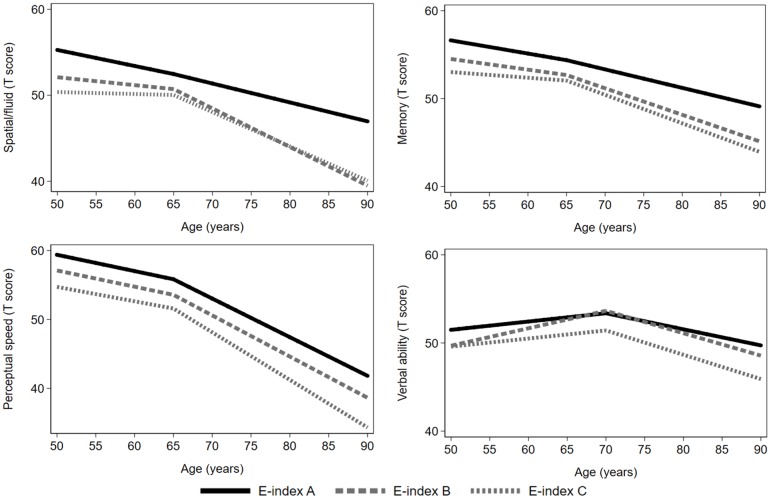
**Age–related cognitive trajectories in different domains by Eichner categories.** Model with age as timescale, adjusted for sex, education, birth cohort, and practice effects (n=544). The reference group was Eichner category A. A knot was placed at age 65 (spatial/fluid abilities, memory, and perceptual speed) or at age 70 (verbal ability).

In basic-adjusted model (sex, education, birth cohort, and practice effects), compared to the participants in Eichner category A (optimal masticatory ability), those in category B had a lower performance in verbal ability at intercept. Moreover, participants in category B and C had a steeper decline in spatial/fluid abilities after age 65. There was no significant difference between Eichner category A relative to B or C in the intercept or slopes for perceptual speed, memory or the cognitive component score (Table 2 and Figure 1). After further adjustment for hypertension, heart disease, periodontal disease, prosthesis use, childhood SES, and alcohol consumption, the association between Eichner category B and verbal ability intercept remained significant, as did the association between Eichner category B and C with spatial/fluid abilities slope after age 65 (Supplementary Table 1).

**Table 2:** β-coefficients and 95% confidence intervals (CI) for age-related differences in mean cognitive performance and decline in different domains (T-scores) in relation to the Eichner Index (*n*=544).

	**Eichner Index**	**Spatial/fluid abilities**	**Verbal ability**	**Memory**	**Perceptual speed**	**Component score^a^**
		**β (95% CI)**	**β (95% CI)**	**β (95% CI)**	**β (95% CI)**	**β (95% CI)**
Intercept	**A** (Ref)	68.23 (60.39 to 76.08)	48.69 (43.79 to 53.59)	60.45 (51.01 to 69.88)	68.37 (62.01 to 74.72)	62.19 (56.88 to 67.51)
	**B**	-6.90 (-17.40 to 3.60)	-6.94 (-12.48 to -1.40)	-4.59 (-18.49 to 9.31)	-1.79 (-13.67 to 10.08)	-3.23 (-11.56 to 5.10)
	**C**	-10.55 (-25.53 to 4.43)	-1.95 (-9.73 to 5.83)	-7.48 (-25.81 to 10.83)	-6.09 (-16.49 to 4.31)	-3.07 (-12.03 to 5.90)
Slope (linear age up to 65)^b^						
	**A** (Ref)	-0.19 (-0.31 to -0.06)	0.09 (0.04 to 0.15)	-0.14 (-0.29 to 0.01)	-0.24 (-0.34 to -0.14)	-0.14 (-0.22 to -0.06)
	**B**	0.07 (-0.11 to 0.24)	0.10 (0.02 to 0.18)	0.01 (-0.22 to 0.23)	-0.01 (-0.20 to 0.19)	0.02 (-0.11 to 0.16)
	**C**	0.10 (-0.13 to 0.34)	-0.00 (-0.11 to 0.11)	0.07 (-0.22 to 0.36)	0.03 (-0.14 to 0.20)	-0.01 (-0.15 to 0.13)
Slope (linear age from 65)^b^						
	**A** (Ref)	-0.30 (-0.39 to -0.21)	-0.18 (-0.26 to -0.11)	-0.21 (-0.31 to -0.10)	-0.56 (-0.65 to -0.46)	-0.36 (-0.43 to -0.28)
	**B**	-0.16 (-0.30 to -0.03)	-0.08 (-0.19 to 0.05)	-0.09 (-0.24 to 0.05)	-0.04 (-0.18 to 0.09)	-0.04 (-0.17 to 0.10)
	**C**	-0.15 (-0.28 to -0.02)	-0.10 (-0.21 to 0.01)	-0.12 (-0.26 to 0.03)	-0.14 (-0.27 to 0.00)	-0.10 (-0.27 to 0.04)

Model with age as timescale, adjusted for sex, education, birth cohort, and practice effect. The reference group was Eichner Index A.

^a^Cognitive component of tests for spatial/fluid abilities, verbal fluency, memory, and perceptual speed.

^b^A knot was placed at age 65 for spatial/fluid, memory, and perceptual speed and at age 70 for verbal ability.

### Eichner categories in relation to dementia risk

**Figure 2:**
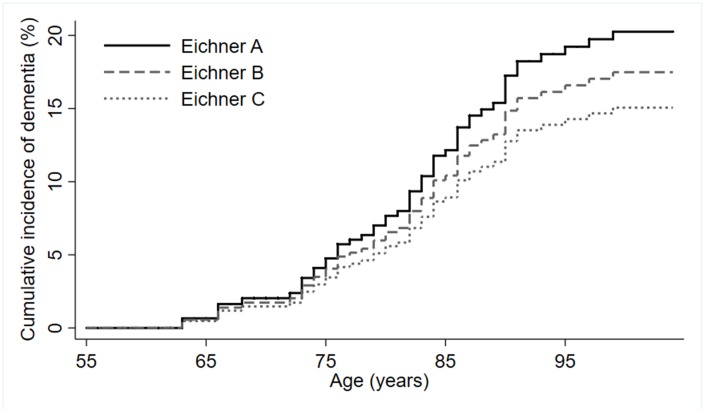
**Cumulative incidence of dementia by Eichner categories (*n*=544).**

During follow-up time (median=10 years, IQR= 16-3), accounting for a total of 8638 person-years, 52 out of the 544 (9.6%) participants developed dementia (8.6 cases per 1000 person-years). In crude and adjusted Cox regression models, estimates did not indicate higher dementia risk for participants in Eichner categories B and C compared to Eichner A (Table 3). Figure 2 shows the cumulative incidence of dementia according to Eichner categories accounting for the competing risk of death.

**Table 3:** Incidence rates (IR) per 1000 person-years and hazard ratios (HR) with 95% confidence intervals (95% CI) of all-cause dementia (*n*= 52) over 22-year follow-up by Eichner categories.

**Eichner Index**	**No. events/ person-years**	**IR (95% CI)**	**HR (95% CI)^a^**	**Adjusted HR (95% CI)^b^**	**Multi-adjusted HR (95% CI)^c^**
A	13/2496	5.20 (3.02 to 8.97)	1.00 (reference)	1.00 (reference)	1.00 (reference)
B	17/2420	6.91 (4.29 to 11.11)	0.90 (0.43 to 1.86)	0.83 (0.39 to 1.76)	1.03 (0.43 to 2.44)
C	22/2957	7.44 (4.90 to 11.30)	0.73 (0.35 to 1.50)	0.63 (0.30 to 1.29)	0.79 (0.31 to 2.03)

^a^Crude model.

^b^Model adjusted for baseline sex and education.

^c^Additionally adjusted for birth cohort, hypertension, heart disease, periodontal disease, childhood SES, prosthesis use, diabetes, cerebrovascular disease, and alcohol consumption.

## DISCUSSION

In this population–based longitudinal study with up to 22 years of follow-up, we found that having less posterior occlusal support, a marker of poorer masticatory ability, was related to the level of verbal ability and faster rate of decline in spatial/fluid abilities after the age of 65. We did not find an association between Eichner category and the risk of dementia.

In recent years, several studies have investigated the link between oral health and cognition in older age [21]. Tooth loss was used as a marker of oral health in most of these studies. A majority showed an association between tooth loss and cognitive decline [4, 5]. However, it is possible that some long-term adaptation to the edentulousness/tooth-loss condition in mastication is possible. Therefore, although being practical and reliable, the number of teeth lost per se may not fully reflect masticatory function. Using self-reported masticatory ability as an exposure of interest, a cross-sectional Swedish study of older community dwelling people showed that participants with difficulty in chewing hard food such as apples had a lower cognitive performance [22]. The authors also found that whether or not the dentition was natural or prosthetic had no significant influence on the observed association, suggesting that in mastication, the posterior teeth, more importantly, the contact of these teeth plays the most important role in maintaining cognition. This was indeed supported by our findings, whereby having more posterior contact was associated with better cognitive abilities in older Swedish adults over up to 22 years.

Previous research conducted on the relationship between mastication and cognition has been limited to cross-sectional studies, therefore the temporality for the observed associations is unclear. Some studies have focused on occlusal contacts as a measure of masticatory ability, which has been shown to correspond to self-reported and functional assessments of mastication [23]. In cognitively normal older Dutch persons, mastication was assessed by maximum mouth opening, jaw movement, bite force and the number of occluding pairs. The study revealed that 19% of the variation in episodic memory function could be predicted by jaw mobility and bite strength and that 22% of the variation in executive function was related to self-reported complaints about masticatory function [11]. Another study showed that word recall, verbal fluency, and numeracy was significantly better in people with good chewing ability, functionally measured with a two-color gum mixing ability test [24]. Two cross-sectional studies showed an association between masticatory ability and verbal fluency [24, 25].

In our study, we found baseline associations between Eichner categories and verbal ability, however, we found no effects of masticatory ability on trajectories of change in verbal ability. This could be because the long-term effects of masticatory problems have different effects as opposed to short term. Verbal ability at any one moment could be affected by mobility problems of the jaw as well as pronunciation difficulties due to more recent tooth loss, rather than a manifestation of neural abnormalities related to poor oral health [26]. Nevertheless, the association between Eichner categories and performance in any cognitive domain at intercept should be interpreted with caution, as there were very few data points available at age 50 in this sample. Similarly, we did not find an association between mastication and memory, as two previous cross-sectional studies have shown [11, 27]. The reason for this could be reverse causality due to pre-clinical cognitive impairment or dementia [28], which can lead to poorer oral health care. When examining the cognitive domains in relation to posterior occlusal support in our study, we removed participants with CIND at baseline, and examined the effects of mastication on normal aging.

Thus far, only two studies examined the relationship between masticatory and cognitive function or dementia longitudinally. One study reported a steeper decline in those with fewer posterior occlusal pairs [20]. However, cognitive function was measured using the Japanese version of the Montreal Cognitive Assessment, and therefore there was no indication of the longitudinal effects of mastication on specific cognitive domains, which could elucidate potential mechanisms involved. In the present study, we found an association between occlusal support and spatial/fluid ability. This is in line with a cross-sectional study showing that poorer mastication is related to worse executive functioning as well as reduced cerebral blood flow to the pre-frontal cortex, responsible for higher order cognitive processes [14]. There are several plausible mechanisms explaining the association between occlusal support and accelerated cognitive decline. A loss of posterior occlusal support encompasses a reduction in afferent nerve stimulation, which may cause sensory and motor cortical reorganization [29–31], affect cerebral functional streams toward multisensory hubs [32, 33], and result in memory and cognitive impairment [20]. Reduced masticatory stimulation might lead to cognitive decline through decreases in cerebral blood flow, decreases in activation of the cortical area and blood oxygen levels, particularly to the frontal cortex [12, 13].

It is important to note the selective survival in this study. Those who died had higher proportions of belonging to Eichner categories B and C, had lower baseline cognitive function and were older and in overall worse health. This means that our results may have been underestimated due to selective survival. Nevertheless, this study has a very long follow-up of maximum 22 years, and the mean age at baseline was 63, therefore, it is expected that a high proportion will die during such a long follow-up. As the results showed that Eichner B and C categories were associated with lower cognitive ability, it is possible that if the participants who died had been included, the association would have been even stronger.

To the best of our knowledge, this is the first prospective study that has investigated the association between an objective measure of mastication and the risk of dementia. We did not find poorer masticatory ability to be associated with a higher risk of dementia. This is in line with another study showing no increased risk of dementia over 4 years in participants with poorer self-reported mastication [34]. However, on average, participants with Eichner C were 10 years older than those in category A, and were overall in worse health and had the highest proportions of death during the study period. Therefore, while poor masticatory function could accelerate cognitive decline, due to the likely competing risk of death, those with the poorest masticatory function may not live long enough to develop clinical manifestations of dementia. Nevertheless, due to the low number of dementia incidence cases, the analysis of the association between Eichner categories and dementia risk may have been underpowered.

The major strengths of our study are the population–based cohort design, the long follow–up time, and the repeated cognitive testing. Furthermore, using composite scores of cognitive domains reduces ceiling and floor effects and measurement error variance common in single cognitive tests. However, some limitations need to be pointed out. First, the statistical power to detect group differences in stratified analyses regarding relevant population characteristics such as birth cohort, education and socioeconomic status was insufficient due to limited sample sizes. Second, we could not account for other functionally important factors involved in mastication such as pain, salivation or jaw mobility, which could have led to an underestimation of the current results.

In conclusion, this study provides evidence that poorer posterior occlusal support as a measure of masticatory ability is associated with a faster age-related decline in in spatial/fluid abilities. Further longitudinal studies with larger sample sizes exploring the association between mastication and cognitive health are warranted.

## MATERIALS AND METHODS

### Study population

The Swedish Adoption/Twin Study of Aging (SATSA) is a population–based longitudinal study consisting of a subset of participants from the Swedish Twin Registry (STR) [35]. The study design of SATSA has been reported in detail elsewhere [36]. Briefly, from the base population of SATSA, all twin pairs who were aged ≥50 and participated in a mailed questionnaire in 1984 were invited to undergo clinical examinations and cognitive assessments by trained nurses starting in 1986 (first in–person testing, IPT1; *n*=759). Subsequently, the participants were followed-up every three years from 1986 until 2012. Throughout the study period, nine waves of examinations (IPT1 to IPT9) were carried out. Information on dental status was collected during IPT2 (1989-1991), therefore, only the participants who were assessed at IPT2 were included in this study (*n*=595), henceforth referred to as the baseline. After excluding participants with missing information on dental status (*n* = 5), with dementia (*n* = 8) or cognitive impairment no dementia (CIND) at baseline (*n*=38), 544 dementia-free participants remained for the current study.

Informed consent was obtained from all participants. SATSA was approved by the Regional Ethics Board at Karolinska Institutet, Stockholm, Sweden. Confidentiality and anonymity were guaranteed as part of the informed consent. Participants were informed that their involvement in the study was voluntary and that they were free to withdraw from the study at any point in time.

### Data collection

From the original questionnaire in 1984, information was collected on demographics (i.e., education) and at each wave, on lifestyle factors (i.e., smoking, alcohol consumption, and physical exercise). Nurses measured blood pressure, weight, and height at baseline and at each follow–up examination. Information on medical conditions (e.g. hypertension, heart diseases), and medication use was obtained through self–report at baseline and each follow-up examination. Specifically, hypertension was defined as resting blood pressure ≥140/90 mmHg, and/or self-reported use of antihypertensive medication [37]. Heart diseases (heart failure, coronary heart diseases, and heart attack), and stroke, and were assessed based on self-reported medical history at baseline. Diabetes was ascertained at baseline and each follow-up based on self-reported medical history, use of hypoglycaemic medications (oral hypoglycemic agents or insulin), or FBG ≥7.0 mmol/L, or nonfasting blood glucose (noFBG) ≥11 mmol/L. Blood samples were taken at study entry and the apolipoprotein E (*APOE*) gene was genotyped utilizing high-throughput sequencing and dichotomized as any ε4 carriers or ε4 noncarriers.

Educational level was dichotomized as low (elementary or vocational, ≤ 9 years) and high (high school or above, >9 years). Body mass index (BMI) was calculated as weight in kilograms divided by squared height in meters (kg/m2) [38]. Smoking status was categorized as non‒smoker (participants who had never smoked), past smoker and current smoker. Alcohol consumption was dichotomized as never-drinker (never drink alcohol) and drinker (former and current drinker). Socioeconomic status (SES) in childhood (rearing home) was measured from a scale including three components: material resources within the household, highest education of the parents, and highest occupational status of the parents. This scale is based on factor analyses. Variables were standardized to a mean of 0 and a standard deviation of 1 before summing. A higher score on the scale reflects higher SES level [39].

### Assessment of dental status

During IPT2 trained nurses examined and recorded the presence/absence of each tooth and type of filling if present. The nurses also collected information using a questionnaire about whether the participants used prostheses, categorized as none, half-prosthesis or whole prosthesis. Additionally, the participants were asked if they have problems with gingivitis (bleeding gums; no/yes or sometimes) or periodontal disease (yes/no).

Each participant was categorized according to the Eichner Index [18]. In the Eichner classification, each posterior contact, including both the premolar and molar regions, are counted as one zone, yielding a total of four supporting zones [40]. The Eichner Index describes the existing posterior occlusal support zones by dividing the occlusal status into three main groups (A, B and C). Individuals classified in Group A have occlusal contacts in all four posterior support zones (indicating optimal masticatory ability), those in group B have 1-3 occlusal contacts (indicating moderate masticatory ability) and those in group C have no posterior occlusal contact at all (indicating poor masticatory ability). The categorization was checked for errors independently by I.W. (DDS, specialist in Orofacial medicine) for a random 10% of the sample.

### Assessment of cognitive domains, CIND, and dementia

The cognitive battery included 12 tests assessing four cognitive domains: verbal abilities (information, synonyms, and analogies), spatial/fluid (Figure logic, Kohs Block Design, and Card rotations), memory (Digit span forwards and backward, Thurstone’s pictures memory, Name and faces immediate and delayed recall), and perceptual speed (Symbol digit, and Figure identification) [41]. These domains were identified by principal–component analysis (PCA) [42]. Briefly, cognitive assessments at each wave were standardized using the means and variances observed at baseline. For each wave, a factor representing each cognitive domain was generated by combining the standardized cognitive scores using the factor weights derived from the PCA at baseline. A cognitive component was created based on the first principal component of nine cognitive tests (Information, Synonyms, Analogies, Koh's Block Design, Card Rotations, Thurstone's Picture Memory, Digit Span forwards and backward, Symbol Digit, and Figure Identification). All component scores were rescaled as t-scores by adding a constant of 50 and multiplying by 10 [43].

Cognitive impairment–no dementia (CIND) was considered as the condition where the observed cognitive deficits were not severe enough to meet the criteria for dementia diagnosis. A person was categorized has having CIND if the person’s Mini–Mental State Examination (MMSE) at study entry was at least 1 SD or 2 SDs below the age- and education-specific mean MMSE in people aged 50-75 years or ≥75 years, respectively [44]. Dementia was diagnosed at follow–up examinations according to criteria from the *Diagnostic and Statistical Manual of Mental Disorders, Third or Fourth Editions* (*DSM–III* or *DSM–IV*) [42, 45]. Clinical diagnosis of dementia was determined during a consensus meeting, in which performance on cognitive tests, health, daily functioning, and medical records were reviewed [46].

### Statistical analyses

Differences in characteristics of the participants by Eichner categories (A, B, and C) at study entry were assessed using chi-square (χ2) or two–tailed one-way ANOVA with post hoc group comparisons with Bonferroni correction. Piecewise linear mixed-effects models were used to estimate the association of Eichner categories with intercept and annual rate of change in each cognitive domain using age as the time scale. Previous studies on the SATSA cohort have shown that age-related decline in memory, spatial ability, and perceptual speed starts around age 65, therefore a knot was positioned at age 65 for these domains, whereas for verbal ability decline starts around 70 hence, the knot was positioned at age 70 [47]. The fixed effects included baseline Eichner category (category A vs category B or C), linear age, and their interaction (Eichner category × age). All models included a random intercept and two random slopes (splines) for age before and after the knot, allowing individual differences at intercept and over time (age). The random effects accounted for both the repeated measures for each person and the presence of twin pairs by using a person–specific identifier and a common twin–pair identifier. The follow–up was censored when dementia occurred. An unstructured variance–covariance matrix was employed in all models with robust standard errors. Likelihood-ratio tests were used to determine which parameters should be included in the final model.

Sex, education, and birth cohort (defined as early birth Cohort 1: born 1886-1925 and late birth Cohort 2: born 1926-1958) were included as covariates in the main analysis. Birth cohort was adjusted for as the participants had a wide age-range at study entry, which may give rise to cohort effects due to the long follow-up period. To account for the possibility of practice effects for the cognitive testing, we also included a time-varying retest covariate (“First cognitive assessment” vs “Follow-up assessment”). In additional analyses, we further adjusted for demographic factors, baseline lifestyle factors and medical conditions such as alcohol consumption, hypertension, heart disease, prosthesis use, periodontal disease, cerebrovascular disease, diabetes, and childhood socioeconomic status.

Incidence rates (IRs) and 95% confidence intervals (CIs) of dementia per 1000 person-years were calculated for participants as the number of events during the follow-up period divided by person-years of follow-up. Cox proportional hazards regression models were used to estimate the cause-specific hazard ratios (HRs) and 95% CIs of dementia related to Eichner category A, B or C. Follow-up time was calculated as age from study entry until dementia diagnosis, otherwise until death or last in-person testing. Age was used as the time scale. The proportional hazard assumption was tested for the predictor and covariates, using Schoenfeld’s residuals regressed against follow-up time (age). No violation of proportionality was observed. Crude, basic-adjusted (sex and education), and multi-adjusted models (additionally adjusted for birth cohort, childhood socioeconomic status, periodontal disease, hypertension, heart disease, alcohol use and prosthesis use) were computed. A robust standard error estimator was used to adjust for the potential dependencies within twin pairs [48]. All statistical analyses were performed with Stata SE 15.0 (StataCorp LP., College Station, Texas, USA) and RStudio v. 1.2.5001 (RStudio: Integrated Development for R. RStudio, Inc., Boston).

## Supplementary Material

Supplementary Table 1
