# Supplementary material for: The relation of poor mastication with cognition and dementia risk: a population-based longitudinal study
Source: Aging (Albany NY). 2020 Apr 30;12(9):8536–48. doi: 10.18632/aging.103156 (PMC7244038; doi:10.18632/aging.103156)
Supplement: Supplementary Table 1 [file aging-12-103156-s001..pdf]

SUPPLEMENTARY TABLE

Supplementary Table 1. Multi-adjusted age-related differences in mean cognitive performance and decline (95% Confidence Intervals) in different domains by Eichner Index (*n*=544).

	Eichner Index	Spatial/fluid abilities	Verbal ability	Memory	Perceptual speed	Component score*
		β (95% CI)	β (95% CI)	β (95% CI)	β (95% CI)	β (95% CI)
Intercept	A (Ref.)	64.26 (55.56 to 72.96)	46.00 (41.00 to 51.00)	60.47 (49.75 to 71.18)	69.40 (62.40 to 76.40)	67.38 (58.90 to 75.85)
	B	-6.15 (-16.95 to 4.65)	-8.80 (-14.51 to -3.09)	-8.99 (-24.00 to 6.00)	-1.80 (-14.10 to 10.50)	-5.84 (-14.24 to 2.56)
	C	-14.07 (-28.42 to 0.27)	1.12 (-7.64 to 9.89)	1.00 (-20.50 to 22.50)	-0.13 (-13.55 to 13.29)	4.16 (-10.28 to 18.59)
Slope (linear age up to 65)**						
	A (Ref.)	-0.15 (-0.28 to -0.02)	0.08 (0.02 to 0.13)	-0.18 (-0.35 to -0.02)	-0.24 (-0.34 to -0.14)	-0.18 (-0.27 to -0.10)
	B	0.06(-0.11 to 0.24)	0.13 (0.05 to 0.21)	0.12 (-0.12 to 0.36)	-0.02 (-0.22 to 0.19)	0.06 (-0.08 to 0.20)
	C	0.16 (-0.07 to 0.39)	-0.05 (-0.17 to 0.08)	-0.05 (-0.38 to 0.29)	-0.08 (-0.29 to 0.14)	-0.12 (-0.35 to 0.11)
Slope (linear age from 65)**						
	A (Ref.)	-0.27 (-0.37 to -0.18)	-0.20 (-0.26 to -0.12)	-0.24 (-0.35 to -0.12)	-0.61 (-0.70 to -0.51)	-0.44 (-0.53 to -0.35)
	B	-0.16 (-0.30 to -0.02)	-0.07 (-0.19 to 0.04)	-0.12 (-0.27 to 0.03)	0.01 (-0.13 to 0.15)	-0.01 (-0.15 to 0.12)
	C	-0.15 (-0.30 to -0.01)	-0.10 (-0.21 to 0.01)	-0.12 (-0.27 to 0.04)	-0.10 (-0.24 to 0.04)	-0.12 (-0.27 to 0.03)

Model adjusted for sex, education, practice effect, birth cohort, hypertension, heart disease, *APOE* ε4, periodontal disease, childhood SES, prosthesis use, diabetes, cerebrovascular disease, and alcohol consumption. The reference group was Eichner Index A (optimal masticatory ability).

* Component score of tests for spatial/fluid abilities, verbal ability, memory, and perceptual speed.

** A spline was placed at age 65 for spatial/fluid abilities, memory, and perceptual speed and at age 70 for verbal ability.
